# Clinical characteristics and etiological analysis of deep fungal infections in a general hospital in Southwest China (2020–2024)

**DOI:** 10.3389/fpubh.2026.1766256

**Published:** 2026-04-01

**Authors:** Shuwei Zhao, Rui Su, Zhenghui Yang, Yuye Li, Yi-Qun Kuang, Hongbin Li

**Affiliations:** 1 Department of Dermatology and Venereology, First Affiliated Hospital of Kunming Medical University, Kunming, China; 2 Research Center for Clinical Medicine, First Affiliated Hospital of Kunming Medical University, Kunming Medical University, Kunming, China

**Keywords:** Aspergillus, biological agents, deep fungal infection, epidemiology, prognosis, risk factors

## Abstract

**Background:**

Deep fungal infections (DFIs) represent a significant and growing threat to hospitalized patients, contributing to substantial morbidity and mortality worldwide. The healthcare landscape has undergone notable changes in recent years, particularly with the impact of the COVID-19 pandemic, which has altered host immunity and care pathways, potentially influencing the epidemiology of DFIs.

**Objective:**

This study aimed to delineate the clinical characteristics, mycological profiles, risk factors of deep fungal infections, and to evaluate factors associated with mortality among inpatients in a general hospital in southwestern China from 2020 to 2024.

**Methods:**

This was a retrospective analysis of 886,056 patients in the hospital from 2020 to 2024, including the epidemiological characteristics, treatment, and prognosis of deep fungal infections.

**Results:**

A total of 462 cases (0.052%) with deep fungal infections were identified, accounting for 0.052% of the total number of hospitalizations. The incidence of deep fungal infections in the hospital showed an increasing trend from 2020 to 2024. Compared with previous studies, *Candida* remained the predominant pathogen (84.20%), with increased proportions of *Aspergillus* (13.90%) and *Cryptococcus* (6.71%). A new case of *Mucor* infection (0.22%) was reported. The respiratory tract remains the most common site of infection (88.60%). In terms of treatment, most patients received azole therapy (88.30%), with those treated with echinocandins showing significantly higher survival rates (*P* = 0.033). Multiple logistic regression analyses revealed that mixed infections, hematologic diseases, and biological agents were significant risk factors for poor prognosis (all *P* < 0.05).

**Conclusions:**

Compared with earlier research (2015–2019), the number of deep fungal infection cases from 2020 to 2024 has shown a significant and sustained increase. Moreover, the threat posed by *Aspergillus* and *Cryptococcus* has increased. Although clinical data remain limited, these findings provide valuable insights for the prevention, diagnosis, and treatment of deep fungal infections. Compared with earlier research (2015–2019), the number of deep fungal infection cases from 2020 to 2024 has shown a significant and sustained increase. Moreover, the threat posed by *Aspergillus* and *Cryptococcus* has increased. Although clinical data remain limited, these findings provide valuable insights for the prevention, diagnosis, and treatment of deep fungal infections.

## Introduction

Deep fungal infections are a growing global public health concern. Every year, more than 650 million people worldwide are affected by fungal infections, of which more than 7 million cases are severe invasive fungal diseases, resulting in approximately 1.35 million deaths (1). The occurrence of infection is closely related to the application of broad-spectrum antibiotics, immunosuppressants, catheter technology, surgery, long-term hospitalization, and glucocorticoids, as well as to severe underlying diseases such as organ transplantation and hematologic malignancies (2–4). The coronavirus disease 2019 (COVID-19) pandemic has led to unprecedented alterations. Substantial evidence suggests that critically ill COVID-19 patients, due to virus-induced endotheliopathy and immune dysregulation, coupled with immunomodulatory therapies, constitute a newly recognized high-risk cohort for both candidaemia and COVID-19-associated pulmonary aspergillosis (CAPA) (5–7).

The primary pathogens causing deep fungal infections in humans can be categorized into two major groups: yeasts and filamentous fungi. Among yeasts, *Candida* species are predominant, with over 90% of invasive candidiasis cases attributed to five common pathogens: *Candida albicans* (*C. albicans*), *Candida glabrata* (*C. glabrata*), *Candida tropicalis* (*C. tropicalis*), *Candida parapsilosis* (*C. parapsilosis*), and *Candida krusei* (*C. krusei*) (8). *C. albicans* is the most common pathogen, but the proportion of nonalbicans cases has increased in recent years, especially among patients in intensive care units (ICUs) (9–11). *Aspergillus* is the primary pathogen of filamentous fungal infections. It can cause invasive pulmonary aspergillosis and is more common in patients with hematologic malignancies and those who are critically ill. The incidence of *Mucor* is lower than that of *Aspergillus*, but the disease is severe, and mortality is very high, which is common in immunosuppressed patients (10, 12, 13).

Ran et al. previously described the pathogenic microbial characteristics, clinical features, and risk factors for patients with deep fungal infections in general hospitals in Southwest China from 2015 to 2019, establishing an essential epidemiological baseline for this study (14). Therefore, this study aims to further explore the status of deep fungal infection in the post COVID-19 pandemic era through a retrospective analysis of deep fungal infection cases in the region from 2020 to 2024, analyze the differences in prognosis among patients with deep fungal infections, further investigate the independent risk factors associated with prognosis, and provide a practical foundation for prognostic evaluation in this patient population.

## Materials and methods

### Patient selection

We screened inpatients from January 2020 to December 2024 from the medical record room of the First Affiliated Hospital of Kunming Medical University, and then screened out cases of fungal infection based on their laboratory tests. Each hospitalization represented a case. A new independent case was defined only when all three of the following criteria were met: (i) an interval of more than 3 months between hospitalizations; (ii) isolation of a different fungal species; and (iii) administration of a new course of antifungal therapy. The case exclusion criterion was cases with incomplete data on epidemiology, fungal microscopy, and culture examinations.

### Criteria for study inclusion

A retrospective investigation method was used to retrospectively analyze 886,056 medical records from the inpatient department of the First Affiliated Hospital of Kunming Medical University from January 2020 to December 2024, and to perform statistical analysis on the cases of fungal infection via laboratory tests. The diagnostic criteria for patients with deep fungal infections included in this study were based on the diagnostic criteria for nosocomial infections, EORTC/MSGERC (15). The analyzed information included general details about the patient, the underlying disease, fungal infection, and the use of antifungal medications.

### Methods for identifying fungal agents

Fungal identification was performed following the recommended diagnostic algorithm for invasive fungal infections (16, 17).

Specimen processing and culture. Clinical specimens, including sputum, bronchoalveolar lavage fluid, blood, cerebrospinal fluid, and sterile site exudates, were inoculated onto Sabouraud dextrose agar supplemented with chloramphenicol (0.05 g/L) to inhibit bacterial growth. Cultures were incubated at 35 C for 5–7 days. Positive cultures were subcultured onto fresh Sabouraud dextrose agar and blood agar to obtain pure colonies for further identification (18).

Phenotypic identification. Preliminary identification was based on colony morphology, growth rate, pigment production, and microscopic examination using lactophenol cotton blue staining to observe hyphal structure, conidial morphology, and reproductive structures. For yeast isolates, biochemical profiling was performed using the API 20 C AUX system (bioMérieux, France) according to the manufacturer's instructions (19).

MALDI-TOF MS analysis. Filamentous fungi and *Candida* species that are difficult to identify reliably by API 20C AUX (e.g., *C. krusei*) were subjected to MALDI-TOF MS analysis for definitive species identification. Sample preparation was performed using the ethanol-formic acid extraction method (17, 18). Briefly, fungal material was suspended in 300 μL of distilled water and mixed with 900 μL of absolute ethanol, followed by centrifugation. The pellet was air-dried and resuspended in 50 μL of formic acid (70%) and 50 μL of acetonitrile. After centrifugation, 1 μL of the supernatant was spotted onto a target plate, air-dried, and overlaid with 1 μL of α-cyano-4-hydroxycinnamic acid matrix. Spectra were acquired using a Microflex LT mass spectrometer and analyzed with MALDI Biotyper software (version 3.0). Identification scores ≥2.0 were considered reliable for species-level identification, and scores 1.7–2.0 for genus-level identification (17). For most isolates, the combination of phenotypic methods and MALDI-TOF MS provided definitive species identification.

Molecular identification by DNA sequencing. For a subset of isolates where phenotypic or MALDI-TOF MS identification was inconclusive (e.g., ambiguous spectra, low scores, or rare species requiring confirmation), molecular identification was performed by sequencing the internal transcribed spacer (ITS) region of ribosomal DNA, which is the official fungal barcode for species identification (20, 21). Genomic DNA was extracted from pure cultures using the DNeasy Plant Mini Kit (Qiagen, Germany) according to the manufacturer's protocol. The ITS region (including ITS1, 5.8S rRNA, and ITS2) was amplified using universal primers ITS1 (5′-TCCGTAGGTGAACCTGCGG3′) and ITS4 (5′-TCCTCCGCTTATTGATATGC3′) (20). PCR products were purified and sequenced by Sanger sequencing. The resulting sequences were compared against the GenBank database (NCBI) and the UNITE reference database for fungal identification. Species identification was accepted when sequence similarity was ≥99% with reference sequences in the database (21). This molecular approach aligns with the revised EORTC/MSGERC consensus definitions for proven invasive fungal infection, which accept PCR and sequencing from fungal elements as definitive evidence for genus/species identification (21).

### Antifungal treatment regimens

Data on antifungal treatment regimens were collected from medical records, including the specific agents used, dosage, duration, and whether the regimen was administered as monotherapy or combination therapy. Monotherapy was defined as the use of a single antifungal agent, while combination therapy was defined as the concurrent use of two or more antifungal agents from different classes (22). The choice of regimen was at the discretion of the attending physicians based on clinical presentation, identified pathogens, and local treatment protocols.

### Statistical analysis

Statistical analyses were performed via SPSS software (version 26.0). Continuous variables with a normal distribution are presented as the means ± standard deviations, whereas those with a skewed distribution are summarized as medians (interquartile ranges). Categorical variables are reported as numbers (percentages). Group comparisons were conducted via the independent-samples t test, Mann–Whitney U test, or chi-square test, as appropriate. To identify independent risk factors for mortality, variables with a univariate *P* < 0.10 were entered into a multivariable logistic regression model via the forward stepwise selection method (entry: α = 0.05; removal: α = 0.10) (23). The results are expressed as adjusted odds ratios (ORs) with 95% confidence intervals (CIs). A two-sided *P* value of less than 0.05 was considered statistically significant. The coding scheme for all variables included in the logistic regression analysis is summarized in Supplementary Table S1.

## Results

### Incidence rate of deep fungal infections

**Figure 1:**
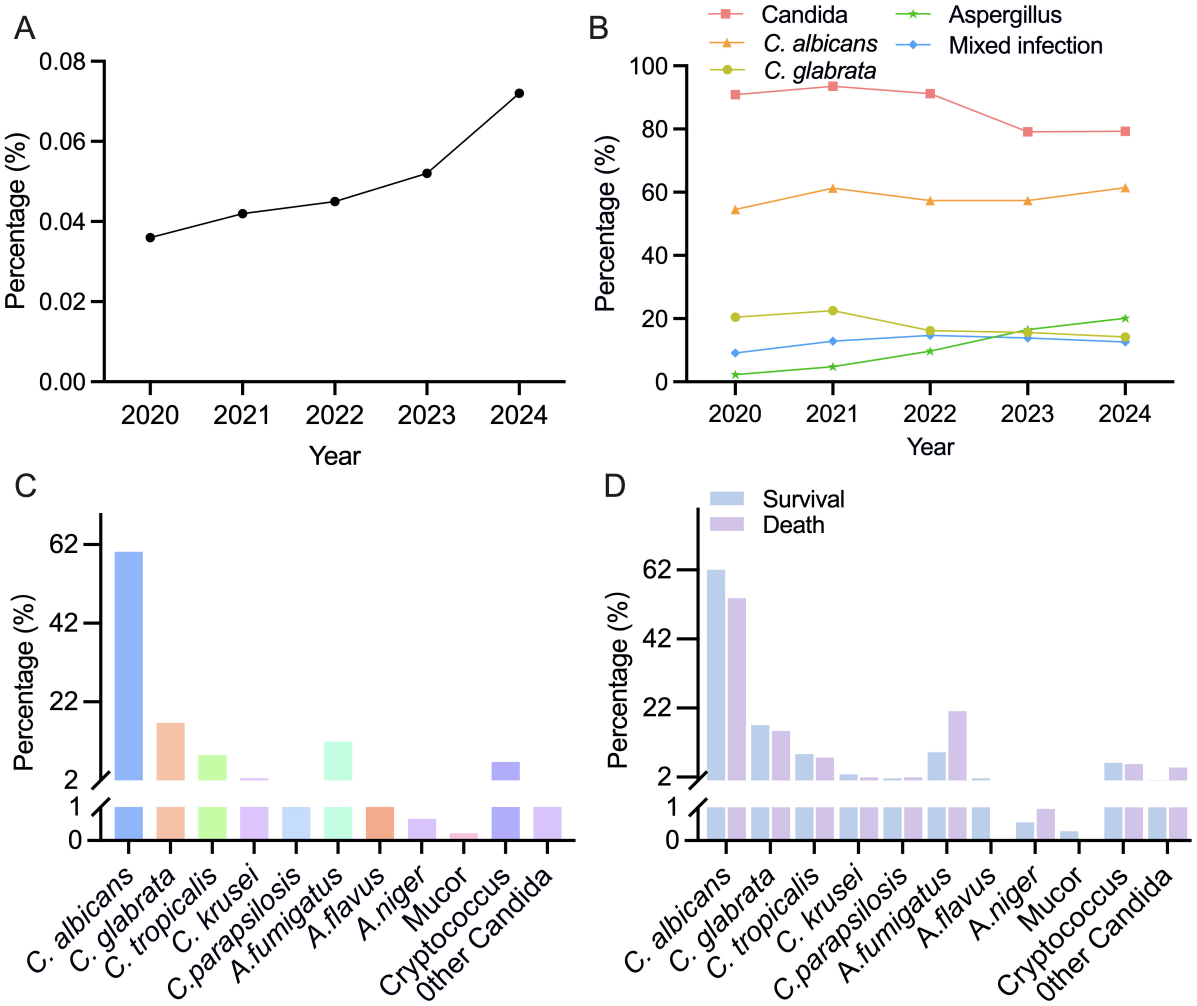
*Pathogens for deep fungal infections and incidence rate*. **(A)** The annual incidence rate of deep fungal infections. **(B)** Temporal trends in the incidence of different fungal strains in deep fungal infections. **(C)** Distribution of pathogenic bacteria in deep fungal infections. **(D)** The intragroup differences in the distribution of pathogenic bacteria.

From January 2020 to December 2024, the First Affiliated Hospital of Kunming Medical University recorded a total of 886,056 inpatient visits, with annual counts of 122,577, 148,636, 150,757, 223,040, and 241,046, respectively. According to the established diagnostic criteria, 462 cases (0.052%) were diagnosed with deep fungal infections, corresponding to 451 unique patients. Among these, 11 patients had repeated admissions that met the criteria for new independent cases. The annual number of cases and corresponding incidence rates from 2020 to 2024, calculated as the number of confirmed deep fungal infection cases divided by the total number of inpatient admissions multiplied by 100%—were 44 (0.036%), 62 (0.042%), 68 (0.045%), 115 (0.052%), and 173 (0.072%), respectively. A retrospective analysis of these data demonstrated a continuous upward trend in the incidence of deep fungal infections over the 5 years, with the highest rate observed in 2024 (Figure 1A).

### Pathogens for deep fungal infections

Over the 5-year study period, the annual incidence of deep fungal infections caused by *Candida* and *Aspergillus* species demonstrated opposing trends. The incidence of *Candida* infections decreased rapidly from 2023 to 2024, dropping from 93.54% to 79.13%. In contrast, *Aspergillus* infections increased steadily throughout the study period, rising from 2.27% in 2020 to 20.11% in 2024. The incidence of mixed fungal infections remained relatively stable, fluctuating between 9.09 and 14.70% annually. Further analysis of the two most prevalent *Candida* species revealed distinct temporal patterns. The proportion of *C.albicans* increased slightly, from 54.54% in 2020 to 61.49% in 2024. Conversely, the proportion of *C.glabrata* exhibited a continuous downward trend, decreasing from 22.58% in 2021 to 14.23% in 2024 (Figure 1B).

Of the 462 cases, *Candida* accounted for 389 cases (84.19%), including *C. albicans* in 278 cases (60.17%), *C. glabrata* in 77 cases (16.67%), *C. tropicalis* in 39 cases (8.44%), *C. krusei* in 12 cases (2.59%) and other *Candida* species in 9 cases (1.95%); *Aspergillus* species accounted for 64 cases (13.9%), including *Aspergillus fumigatus* in 55 cases (11.9%), *Aspergillus flavus* in 6 cases (1.3%), and *Aspergillus niger* in 3 cases (0.65%); *Cryptococcus* was detected in 31 cases (6.71%); and there was one new case of *Mucor* (0.22%; Figure 1C). Mixed infections, defined as the isolation of two or more distinct fungal species from the same patient (24), were observed in 60 cases (12.99%), primarily including dual yeast infections or mixed *Candida*–*Aspergillus* infections.

Compared with the favorable prognosis group, the poor prognosis group presented significantly decreased levels of all yeast pathogens (*P* < 0.01) and significantly increased frequencies of *Aspergillus* infections (*P* < 0.01) and *Cryptococcus* infections. In addition, mixed infections were identified in 20 patients in the mortality group (19.2%), suggesting a significantly greater proportion than those in the survival group (*P* < 0.01; Figure 1D, Table 1).

**Table 1:** General conditions of deep fungal infection patients.

Factors	Survival group (*n* = 358, 77.48%)	Death group (*n* = 104, 22.52%)	Total cases (%)	*P* value^a^
Age (years)	70.00 (19, 4–101)	69.50 (26, 11–97)	70 (21, 4–101)	0.439
Gender				0.774
Male	270 (74.0%)	77 (75.4%)	347 (75.1%)	
Female	88 (26%)	27 (24.6%)	115 (24.9%)	
Hospitalization period (days)	14.00 (11, 3–364)	16.50 (21, 2–700)	14 (13.25, 2–700)	0.053
Infection situation				
Mixed infection	40 (11.2%)	20 (19.2%)	60 (13.0%)	0.031^*^
Candida infections	316 (88.3%)	73 (70.2%)	389 (84.2%)	0.001^*^
Aspergillus infections	41 (11.5%)	23 (22.1%)	64 (13.9%)	0.005^*^
Underlying disease				
Diabetes	42 (11.7%)	20 (19.2%)	62 (13.4%)	0.048^*^
Respiratory diseases	272 (76.0%)	88 (84.6%)	360 (77.9%)	0.062
Cardiovascular diseas	242 (67.6%)	72 (69.2%)	314 (68.0%)	0.753
Hematological diseases	95 (26.5%)	47 (45.2%)	142 (30.7%)	0.001^*^
Malignant tumor	60 (16.8%)	16 (15.4%)	76 (16.5%)	0.739
Neurological diseases	60 (16.8%)	36 (34.6%)	96 (20.8%)	0.001^*^
Renal insufficiency	40 (11.2%)	18 (17.3%)	58 (12.6%)	0.096
Liver dysfunction	47 (13.1%)	16 (15.4%)	63 (13.6%)	0.555
Organ transplantation	9 (2.5%)	4 (3.8%)	13 (2.8%)	0.470
Infectious diseases	13 (3.6%)	3 (2.9%)	16 (3.5%)	0.714
Autoimmune diseases	41 (11.5%)	24 (23.1%)	65 (14.1%)	0.003^*^
Risk factors				
Glucocorticoid	124 (34.6%)	39 (37.5%)	163 (35.3%)	0.591
History of COVID-19 infection	224 (62.6%)	50 (48.1%)	274 (59.3%)	0.008^*^
Immunosuppressant	13 (3.6%)	6 (5.8%)	19 (4.1%)	0.352
Biological agents	13 (3.6%)	13 (12.5%)	26 (5.6%)	0.001^*^
Chemotherapy and Radiotherapy	91 (25.4%)	23 (22.1%)	114 (24.7%)	0.492
Intravenous catheterization	53 (14.8%)	21 (20.2%)	74 (16.0%)	0.187
Treatment				
Combination therapy	58 (16.2%)	22 (21.2%)	80 (17.3%)	0.270
Azoles	318 (88.8%)	90 (86.5%)	408 (88.3%)	0.639
Echinocandins	87 (24.3%)	15(14.4%)	102 (22.1%)	0.033^*^
Amphotericin B	25 (7.0%)	12 (11.5%)	37 (8.0%)	0.132

* *P* < 0.05 was considered statistically significant.

a Continuous variables, such as age and length of hospital stay, were analyzed using the Mann-Whitney U test. Categorical variables were compared using the chi-square test.

All specimens from patients with deep fungal infections yielded positive fungal cultures. A total of 528 fungi isolates were recovered. These included 431 (81.62%) *Candida* isolates, 64 (12.12%) *Aspergillus* isolates, 32 (6.06%) *Cryptococcus* isolates, and one *Mucor* isolate (0.2%). The species distribution, clinical sources, and the identification methods employed are detailed in Table 2.

**Table 2:** Distribution and identification of fungal isolates from patients with DFIs.

Species	No. of isolates (%)	Clinical source (n)	Identification method	Note
*Candida*	431 (81.62%)			
*C. albicans*	282 (54.41%)	*BALF*a**/Sputum (267) Thoraco-abdominal drainage fluid (9) Blood (5) Cerebrospinal fluid (1)	API 20C AUX	Common Candida, API reliable
*C. glabrata*	79 (14.96%)	*BALF*/Sputum (72) Thoraco-abdominal drainage fluid (4) Blood (3)	API 20C AUX	Trehalose(+)
*C. tropicalis*	41 (7.76%)	*BALF*/Sputum (3) Thoraco-abdominal drainage fluid (3) Blood (2)	API 20C AUX	Common Candida, API reliable
*C. krusei*	12 (2.27%)	*BALF*/Sputum (12)	MALDI-TOF MS	API limitations
*C.parapsilosis*	8(1.51%)	*BALF*/Sputum (5) Thoraco-abdominal drainage fluid (3)	API 20C AUX	Common Candida, API reliable
*Kodamaea ohmeri*	4(0.76%)	*BALF*/Sputum (4)	API 20C AUX MALDI-TOF MS	Confirmed by both methods
*Pichia norvegensis*	2(0.39%)	*BALF*/Sputum (2)	MALDI-TOF MS	API cannot identify
*Pichia cactophila*	3(0.57%)	*BALF*/Sputum (3)	API 20C AUX MALDI-TOF MS	Confirmed by both methods
*Aspergillus*	64 (12.12%)			
*Aspergillus fumigatus*	55 (10.41%)	*BALF*/Sputum (55)	Morphology MALDI-TOF MS	Typical morphology, MS confirmed
*Aspergillus flavus*	6 (1.14%)	*BALF*/Sputum (6)	Morphology MALDI-TOF MS	Typical morphology, MS confirmed
*Aspergillus niger*	3 (0.57%)	*BALF*/Sputum (3)	Morphology MALDI-TOF MS	Typical morphology, MS confirmed
*Cryptococcus*	32(6.06%)	*BALF*/Sputum (12) Blood (2) Cerebrospinal fluid (18)	Morphology MALDI-TOF MS	India ink positive, MS confirmed
*Mucor*	1 (0.19%)	*BALF* / Sputum (1)	Morphology ITS^b^ sequencing	Confirmed by molecular method

a *BALF, bronchoalveolar lavage fluid*.

b *ITS, internal transcribed spacer*.

### General information about patients with deep fungal infections

**Figure 2:**
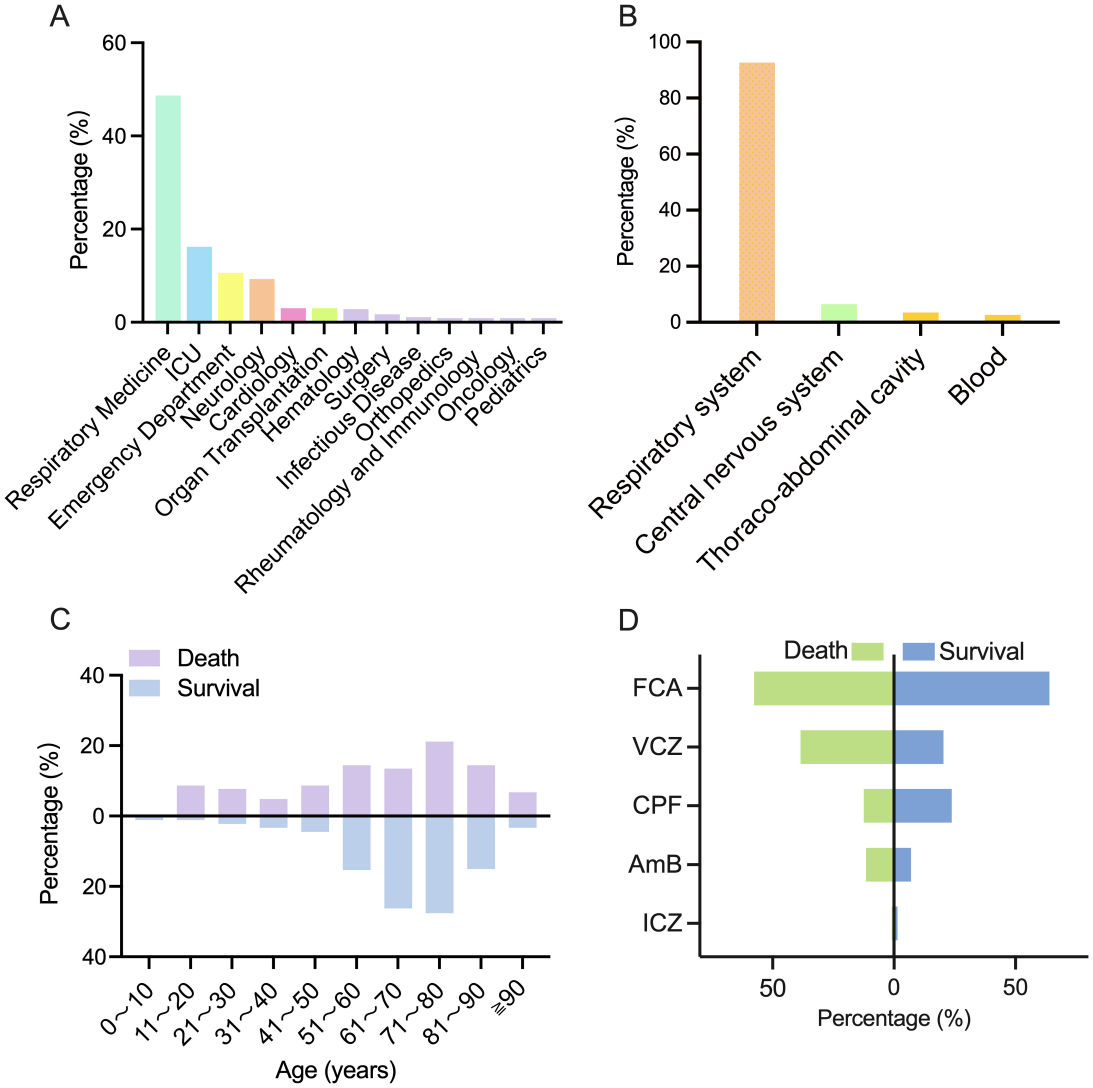
General information about patients with deep fungal infections. **(A)** Distribution of departments for deep fungal infections. **(B)** The distribution of sites for deep fungal infections. **(C)** Age distribution of patients with deep fungal infections. **(D)** Medication distribution and intragroup differences in deep fungal infections.

Among all cases, the Respiratory Medicine was the primary clinical setting, accounting for 225 cases (48.70%), followed by the intensive care unit (ICU) with 75 cases (16.23%) and the Emergency department with 49 cases (10.61%; Figure 2A). Several cases exhibited combined infections at multiple sites, including the respiratory tract and bloodstream. However, the predominant location of deep fungal infection was the respiratory system, accounting for 428 cases (92.64%), followed by the central nervous system, accounting for 19 cases (4.11%); thoracoabdominal drainage fluid, accounting for 16 cases (3.46%); and blood, accounting for 12 cases (2.59%; Figure 2B).

Cases were categorized into survival group and death group based on their clinical results, which included 358 surviving cases (77.48%) and 104 death cases (22.52%). The population characteristics and clinical features are presented in Table 2. The difference in age between the two groups was negligible, with medians and interquartile ranges of 69.50 (26, 11–97) and 70.00 (19, 4–101), respectively. The age distribution revealed that both groups were clustered mainly between 51 and 90 years, with the highest percentages (27.65 and 21.15%) found in the 71–80 age bracket (Figure 2C). The cohort was predominantly male, with 270 (75.4%) in the survival group and 77 (74%) in the death group. The duration of hospitalization was greater in the deceased group: 14.00 (11, 3–364) days compared with 16.50 (21, 2–700) days.

### Risk factors and treatment strategies for deep fungal infections

Most cases with deep fungal infections presented with underlying health issues, primarily respiratory diseases (360 cases, 77.92%), cardiovascular diseases (314 cases, 67.97%), and hematological diseases (142 cases, 30.74%). Respiratory diseases primarily consisted of chronic obstructive lung disease and pneumonia, whereas cardiovascular diseases predominantly included hypertension and coronary heart disease. Cases with diabetes (*P* < 0.05), hematological diseases (*P* < 0.01), neurological diseases (*P* < 0.01), and autoimmune diseases (*P* < 0.01) were much more prevalent in the survival group than in the death group.

Risk factors for deep fungal infections include the use of antibiotics, COVID-19 infection, glucocorticoids, immunosuppressive drugs, biologic agents, chemoradiotherapy, and indwelling catheters. The use of antibiotics was the most common, accounting for 457 cases (98.9%), followed by COVID-19 infection, accounting for 274 cases (59.3%), and glucocorticoid usage, accounting for 163 cases (35.3%), respectively. A statistically significant difference in the use of biologics (*P* < 0.01) and history of SARS-Cov2 infection (*P* < 0.01; Table 2).

All 462 cases received antifungal therapy. Azoles were used in 408 cases (88.30%), including fluconazole in 288 (62.34%), voriconazole in 114 (24.67%), and itraconazole in 6 (1.29%); 98 cases (21.21%) used caspofungin; and 37 cases (8.01%) used amphotericin B (Figure 2D). Combination therapy was offered to 80 cases (17.31%). The amount of Echinocandins used was significantly greater in the survival group (24.3%) than in the death group (*P* < 0.01).

### Independent prognostic factors for HCM patients

Table 3 summarizes the significant risk factors identified through binary logistic regression that are associated with the prognosis of deep fungal infections (*P* < 0.05). Factors positively associated with poor prognosis included mixed infection (OR = 1.893, 95%CI: 1.050–3.408, *P* < 0.05), *Aspergillus* infections (OR = 2.195, 95%CI: 1.247–3.866, *P* < 0.01), hematological diseases (OR = 2.283, 95%CI: 1.453–3.587, *P* < 0.01), neurological diseases (OR = 2.629, 95%CI: 1.611–4.292, *P* < 0.01), autoimmune diseases (OR = 2.320, 95%CI: 1.325–4.062, *P* < 0.01), and use of biological agents (OR = 3.791, 95%CI: 1.699–8.460, *P* < 0.01), whereas *Candida* infection (OR = 0.313, 95%CI: 0.184–0.531, *P* < 0.01), history of COVID-19 (OR = 0.554, 95%CI: 0.357–0.860, *P* < 0.01), and Echinocandin use (OR = 0.525, 95%CI: 0.289–0.955, *P* < 0.01) were identified as negative prognostic factors.

**Table 3:** Results of the binary logistic regression.

Factors	Binary logistic regression			
	β **coefficient**	**OR**	**95% CI**	***P*** **value**^a^
Mixed infections	0.638	1.893	1.050–3.408	0.033^*^
Candida infections	−1.162	0.313	0.184–0.531	0.001^*^
Aspergillus infections	0.786	2.195	1.247–3.866	0.006^*^
Diabetes	0.583	1.791	0.999–3.213	0.051
Hematological diseases	0.825	2.283	1.453–3.587	0.001^*^
Neurological diseases	0.967	2.629	1.611–4.292	0.001^*^
Autoimmune diseases	0.841	2.320	1.325–4.062	0.003^*^
History of COVID-19 infection	−0.591	0.554	0.357–0.860	0.009^*^
Biological agents	1.333	3.791	1.699–8.460	0.001^*^
Echinocandins	−0.644	0.525	0.289–0.955	0.035^*^

* *P* < 0.05 was considered statistically significant.

a *P* values were calculated using the Wald test.

Nine variables with P < 0.10 in binary analysis, including *Candida* infection, history of COVID-19, Echinocandin use, mixed infection, *Aspergillus* infection, hematological diseases, neurological diseases, autoimmune diseases, use of biological agents, and diabetes, were entered into the multivariable logistic regression analysis. Multiple logistic regression analyses revealed that the prognosis with deep fungal infections can be independently predicted by mixed infection (aOR = 0.023, 95%CI: 0.004–0.328, *P* < 0.01), hematological diseases (aOR = 3.314, 95%CI: 1.896–6.145, *P* < 0.01), and the use of biological agents (aOR = 5.128, 95%CI: 1.904–13.810, *P* < 0.01; Table 4).

**Table 4:** Results of the multivariate regression analysis.

Factors	Multivariate regression analysis			
	β coefficient	aOR^a^	95% CI	*P* value^b^
Mixed infections	−3.765	0.023	0.004–0.146	0.001^*^
Hematological diseases	1.228	3.414	1.896–6.145	0.001^*^
Neurological diseases	−0.120	0.887	0.439–1.791	0.737
History of COVID-19 infection	−0.759	0.468	0.184–1.191	0.111
Biological agents	1.635	5.128	1.904–13.810	0.001^*^

* *P* < 0.05 was considered statistically significant.

a adjusted odds ratio obtained from multivariable logistic regression analysis, adjusting for all variables listed in the table.

b *P* values were calculated using the Wald test.

## Discussion

This study reveals a continuous annual increase in the incidence of deep fungal infections (DFIs) at a tertiary hospital in Southwest China from 2020 to 2024, peaking in 2024. This rising trend aligns with global observations during the post-COVID-19 era (5, 6). The pandemic has contributed to a unique ecosystem of hospital-acquired infections, primarily due to virus-induced immune dysregulation and the widespread use of immunomodulatory therapies in critically ill patients (25, 26). Compared to the pre-pandemic data (2015–2019) from the same region by Ran et al. (14), our findings indicate a significant shift not only in incidence but also in the epidemiological landscape of pathogens and patient risk profiles, which warrants further discussion.

The majority of DFI patients in our cohort were elderly median age approximately 70 years and male, which is consistent with the demographic profile of opportunistic infections (27). Notably, while the respiratory department remained the most common infection site, the Intensive Care Unit (ICU) has emerged as the second most common, surpassing transplant departments seen in previous eras (14). This shift underscores the findings of Machado et al. (28), suggesting that the post-pandemic critical care environment has become a central battleground for fungal infections due to the accumulation of multiple risk factors in critically ill patients (29).

Although *C.albicans* remains the dominant pathogen, our study observed a marked shift in the mycological profile. The proportion of *Aspergillus* species reached 13.9% of all cases, representing a more than four-fold increase compared to the 3.01% reported in the 2015–2019 data from the same center (14). This dramatic increase strongly correlates with the global surge in COVID-19-associated pulmonary aspergillosis (CAPA) (6, 28), suggesting that the post-pandemic immunosuppressive environment has created unprecedented opportunities for *Aspergillus* invasion. Furthermore, the increased prevalence of *Cryptococcus* (6.71%) may reflect a growing population with T-cell immune dysfunction, such as those on biologic therapies (4, 30). A new case of *Mucor* infection was also identified, highlighting the expanding spectrum of opportunistic pathogens. Notably, the mortality group showed significantly lower rates of *Candida* infection and significantly higher rates of Aspergillus and mixed infections (*P* < 0.01 for all), suggesting a shift in pathogen profile associated with poor outcome.

Multivariable analysis identified three independent risk factors for poor prognosis in DFI: mixed infections (aoR = 0.023, 95%CI: 0.004–0.146, *P* < 0.001), hematologic diseases (aOR = 3.414, 95%CI: 1.896–6.145, *P* < 0.001), and the use of biological agents (aOR = 5.128, 95%CI: 1.904–13.810, *P* < 0.001). The univariate logistic regression demonstrated that a history of COVID-19 infection was a protective factor for DFI (OR = 0.554, 95%CI:0.357–0.860, *P* < 0.001), with a significantly higher proportion in the survival group (62.6%) than in the death group (48.1%). However, this association did not reach statistical significance in the multivariate regression (aOR = 0.468, *P* = 0.111), suggesting that COVID-19 history is not an independent prognostic factor, and its protective effect may be related to clinical intervention bias for COVID-19 patients during the pandemic (26). The identification of mixed infections as an independent predictor corroborates findings from studies on COVID-19 patients, where polymicrobial infections complicated clinical management and led to higher mortality (31). This association may reflect a state of profound immune dysregulation in the host, making them susceptible to multiple opportunistic pathogens simultaneously. Hematologic diseases emerged as a substantial risk factor. This is likely due to the profound and prolonged neutropenia resulting from both the underlying malignancy and myeloablative therapies, which compromise the host's primary defense against invasive fungi (32, 33). Neutrophils serve as the frontline defense against pathogens such as *Aspergillus* and *Candida*, and their deficiency creates a permissive environment for fungal proliferation. Most notably, our study is the first to confirm in this region that the use of biological agents is the most potent risk factor for a poor prognosis (aOR = 5.128). This finding may be attributable to the limited number of events in the cohort, but it gained considerable international support. A 20-year systematic review by Barbosa et al. (34) confirmed the strong association between TNF-α inhibitors and various invasive fungal diseases. Additionally, Lee et al. (4) reported that psoriasis patients receiving IL-17 inhibitors had a significantly increased risk of invasive aspergillosis, as this pathway plays a key role in anti-*Aspergillus* pulmonary immunity.

While azoles remain the mainstay of antifungal therapy, our data showed that patients treated with echinocandins had significantly higher survival rates. This finding aligns with current clinical guidelines that recommend echinocandins as first-line empirical therapy for suspected invasive candidiasis in critically ill or high-risk patients, particularly in settings where azole resistance is a concern (35, 36). The better outcomes associated with echinocandins in our cohort may be attributed to their excellent tissue penetration (37) and efficacy against azole-resistant *Candida* species, whose prevalence is increasing (38, 39).

This study has several limitations. Due to the retrospective nature of data collection, we were unable to retrieve the original API 20C AUX numerical codes or MALDI-TOF MS scores for individual isolates, as these data were not systematically archived. Molecular confirmation by DNA sequencing was not performed for the majority of isolates, which is a limitation given that DNA-based analysis is considered the gold standard for fungal species identification. Although we employed validated phenotypic methods and selective sequencing for ambiguous cases, the possibility of misidentification cannot be entirely excluded. Future prospective studies with standardized data collection and routine molecular confirmation are warranted to validate our findings.

## Conclusions

In conclusion, this study demonstrates a steadily increasing incidence of deep fungal infections in a Southwest Chinese hospital from 2020 to 2024. The pathogen profile has shifted, with a notable rise in *Aspergillus* and *Cryptococcus* infections alongside persistent Candida dominance. We identified mixed infections, hematologic disorders, and most significantly, the use of biological agents as independent risk factors for poor patient prognosis. Furthermore, initial treatment with echinocandins was associated with improved survival. These findings highlight the evolving challenge of DFIs in the post-pandemic era and underscore the need for heightened clinical awareness, particularly in patients receiving biologic therapies.

## Data Availability

The raw data supporting the conclusions of this article will be made available by the authors, without undue reservation.
