# Supplementary material for: Clinical characteristics and etiological analysis of deep fungal infections in a general hospital in Southwest China (2020–2024)
Source: Front Public Health. 2026 Apr 1;14:1766256. doi: 10.3389/fpubh.2026.1766256 (PMC13080720; doi:10.3389/fpubh.2026.1766256)
Supplement: Supplementary file 1 [file Supplementary_file_1.docx]

**Table S1. Variable coding table**

| Variable | Coding | |
| --- | --- | --- |
| Outcome | 1 = Death | 0 = Survival |
| Mixed infections | 1 = Yes | 0 = No |
| Candida infections | 1 = Yes | 0 = No |
| Aspergillus infections | 1 = Yes | 0 = No |
| Diabetes | 1 = Yes | 0 = No |
| Hematological diseases | 1 = Yes | 0 = No |
| Neurological disease | 1 = Yes | 0 = No |
| Autoimmune disease | 1 = Yes | 0 = No |
| History of COVID-19 infection | 1 = Yes | 0 = No |
| Biological agents | 1 = Yes | 0 = No |
| Echinocandins | 1 = Yes | 0 = No |
